# Synaptic proteins in CSF as potential novel biomarkers for prognosis in prodromal Alzheimer’s disease

**DOI:** 10.1186/s13195-017-0335-x

**Published:** 2018-01-15

**Authors:** Flora H. Duits, Gunnar Brinkmalm, Charlotte E. Teunissen, Ann Brinkmalm, Philip Scheltens, Wiesje M. Van der Flier, Henrik Zetterberg, Kaj Blennow

**Affiliations:** 1 Alzheimer Center and Department of Neurology, Amsterdam Neuroscience, VU University Medical Center, P.O. Box 7057, 1007MB Amsterdam, The Netherlands; 2 Institute of Neuroscience and Physiology, Department of Psychiatry and Neurochemistry, Sahlgrenska Academy at the University of Gothenburg, Mölndal, Sweden; 3 Clinical Neurochemistry Laboratory, Sahlgrenska University Hospital, Mölndal, Sweden; 4 Neurochemistry Laboratory and Biobank, Department of Clinical Chemistry, Amsterdam Neuroscience, VU University Medical Center, Amsterdam, The Netherlands; 5 Department of Epidemiology and Biostatistics, VU University Medical Center, Amsterdam, The Netherlands; 6 Department of Molecular Neuroscience, UCL Institute of Neurology, Queen Square, London, UK; 7 UK Dementia Research Institute at UCL, University College London, London, UK

**Keywords:** Parallel reaction monitoring, Synaptic proteins, CSF biomarkers, Alzheimer’s disease

## Abstract

**Background:**

We investigated whether a panel of 12 potential novel biomarkers consisting of proteins involved in synapse functioning and immunity would be able to distinguish patients with Alzheimer’s disease (AD) and patients with mild cognitive impairment (MCI) from control subjects.

**Methods:**

We included 40 control subjects, 40 subjects with MCI, and 40 subjects with AD from the Amsterdam Dementia Cohort who were matched for age and sex (age 65 ± 5 years, 19 [48%] women). The mean follow-up of patients with MCI was 3 years. Two or three tryptic peptides per protein were analyzed in cerebrospinal fluid using parallel reaction monitoring mass spectrometry. Corresponding stable isotope-labeled peptides were added and used as reference peptides. Multilevel generalized estimating equations (GEEs) with peptides clustered per subject and per protein (as within-subject variables) were used to assess differences between diagnostic groups. To assess differential effects of individual proteins, we included the diagnosis × protein interaction in the model. Separate GEE analyses were performed to assess differences between stable patients and patients with progressive MCI (MCI-AD).

**Results:**

There was a main effect for diagnosis (*p* < 0.01) and an interaction between diagnosis and protein (*p* < 0.01). Analysis stratified according to protein showed higher levels in patients with MCI for most proteins, especially in patients with MCI-AD. Chromogranin A, secretogranin II, neurexin 3, and neuropentraxin 1 showed the largest effect sizes; β values ranged from 0.53 to 0.78 for patients with MCI versus control subjects or patients with AD, and from 0.67 to 0.98 for patients with MCI-AD versus patients with stable MCI. In contrast, neurosecretory protein VGF was lower in patients with AD than in patients with MCI (ß = −0.93 [SE 0.22]) and control subjects (ß = 0.46 [SE 0.19]).

**Conclusions:**

Our results suggest that several proteins involved in vesicular transport and synaptic stability are elevated in patients with MCI, especially in patients with MCI progressing to AD dementia. This may reflect early events in the AD pathophysiological cascade. These proteins may be valuable as disease stage or prognostic markers in an early symptomatic stage of the disease.

**Electronic supplementary material:**

The online version of this article (10.1186/s13195-017-0335-x) contains supplementary material, which is available to authorized users.

## Background

The neuropathological process of Alzheimer’s disease (AD) is characterized by accumulation of plaques composed of aggregated amyloid-β (Aβ) protein, neurofibrillary tangles consisting of hyperphosphorylated tau, and neuronal degeneration and loss [1]. The cerebrospinal fluid (CSF) biomarkers of these processes—amyloid-β 1–42 (Aβ_42_), total tau, and tau phosphorylated at threonine 181 (p-tau)—show very consistent changes in AD dementia and prodromal AD [2], and they have been included as evidence for the presence of AD pathology in research diagnostic criteria for AD [3, 4]. However, in addition to amyloid and tau pathology, processes such as inflammation and synaptic dysfunction also play an important role and may correlate more directly with cognitive decline [5–7]. Hence, biomarkers for these processes may be valuable for disease monitoring and to predict prognosis or rate of cognitive decline.

On the basis of in-house data derived from unbiased CSF proteomic studies, we selected a panel of synaptic and other proteins for analysis with parallel reaction monitoring (PRM) MS. Explorative proteomics provides a hypothesis-free approach for detecting a large number of proteins and peptides in human body fluids such as plasma and CSF [8]; however, the disadvantage of this technique is the relatively poor reproducibility [9]. PRM is an MS method that focuses on predefined sets of proteins, or peptide peaks from proteolytically digested proteins, thereby allowing higher analytical sensitivity and higher throughput of samples [10, 11]. By adding known amounts of isotope-labeled internal standards to the sample, quantification of the actual concentration can be achieved [11]. An advantage over immunoassays is that there is no need for antibodies. Hence, PRM is well suited for validation of explorative proteomic studies [9].

The selection of proteins for the present study was based on a literature review for their possible involvement in AD pathophysiology. The PRM panel consisted of neurosecretory protein VGF (VGF), chromogranin A (CHGA), and secretogranin 2 (SCG2), granins that are presumed to be involved in axonal or synaptic vesicle transport (the granins VGF, CHGA, and SCG2) [12]; cystatin C (CysC), a protease involved in Aβ degradation [13, 14]; β_2_-microglobulin (β_2_M) and lysozyme C (LysC), proteins involved in the innate immune system [15, 16]; and neurexins (NRXNs) NRXN-1, NRXN-2, and NRXN-3 as well as neuronal pentraxin 1 (NPTX1), neurofascin (NFASC), and neurocan core protein (NCANP), proteins involved in synapse formation and stabilization [17–21]. Several of these proteins, including VGF, CHGA, SCG2, CysC, and β_2_M, have been suggested in previous studies to be involved in AD pathology [12–14, 22–28]. A pilot study that we performed after developing the PRM panel showed promising results, with lower levels for several proteins in patients with AD dementia compared with control subjects [29]. The aims of the present study were to validate this panel of proteins in a larger and independent cohort, as well as to investigate whether the panel has potential for distinguishing patients with prodromal AD and patients with AD dementia from cognitively healthy subjects.

## Methods

### Patients

All patients were included, based on the availability of CSF, from the memory clinic-based Amsterdam Dementia Cohort [30]. We selected 40 patients who had received a diagnosis of probable AD dementia and matched them for age and sex with 40 patients with subjective cognitive decline (SCD), who served as control subjects, and with 40 patients with mild cognitive impairment (MCI). All subjects underwent extensive cognitive screening at baseline (between 2004 and 2013), including physical and neurological examinations, electroencephalography, magnetic resonance imaging (MRI), and laboratory tests. Neuropsychological investigation included at least one test per cognitive domain, as well as the Mini Mental State Examination (MMSE) for global cognition. Diagnoses were made by consensus of a multidisciplinary team without knowledge of CSF results. AD dementia and MCI were diagnosed according to the criteria of the National Institute of Neurological and Communicative Disorders and Stroke-Alzheimer’s Disease and Related Disorders Association criteria and Petersen’s criteria, respectively [31, 32], and all patients met the core clinical National Institute on Aging-Alzheimer’s Association criteria [3, 33]. When results of all clinical investigations were normal (i.e., criteria for MCI or any psychiatric or neurological disorder not fulfilled), patients were labeled as having SCD. Patients with MCI were included only when there was clinical follow-up of at least 1 year, and the diagnoses of SCD and AD dementia had to be confirmed at any follow-up visit for subjects to be included in this study. At each follow-up visit, physical, neurological, and neuropsychological examinations were repeated. During follow-up (mean ± SD 2.8 ± 1.1 years), 13 patients with MCI progressed to AD dementia (MCI-AD), 23 patients with MCI remained stable (sMCI), and 3 patients progressed to another type of dementia. All subjects gave written informed consent for the use of their clinical data and CSF for research purposes. The study was approved by the local ethical review board.

### CSF analysis of AD biomarkers

CSF was obtained by lumbar puncture using a 25-gauge needle and a syringe, and it was collected into 10 ml polypropylene tubes (Sarstedt, Nümbrecht, Germany). Part of the CSF was used for routine analysis, including leukocyte and erythrocyte count, glucose concentration, and total protein concentration. Within 2 h, the remaining CSF was centrifuged at 1800 × *g* for 10 minutes at 4 °C, transferred to new polypropylene tubes, and stored either at −20 °C until analysis of Aβ_42_, tau, and p-tau or directly at −80 °C until further analysis. The team involved in CSF analyses was blinded to clinical diagnosis. CSF levels of Aβ_42_, total tau, and p-tau were measured with commercially available enzyme-linked immunosorbent assays (β-amyloid_(1–42)_, hTAU-Ag, and Phosphotau_(181P)_; Fujirebio, Ghent, Belgium) on a routine basis as described elsewhere [34]. Measurements took place consecutively within 1 month of the patient’s baseline visit. Intra-assay coefficients of variation (CVs) were (mean ± SD) 2.0 ± 0.5% for Aβ_42_, 3.2 ± 1.3% for tau, and 2.9 ± 0.8% for p-tau, and interassay CVs (mean ± SD) were 10.9 ± 1.8% for Aβ_42_, 9.9 ± 2.1% for tau, and 9.1 ± 1.8% for p-tau.

### *APOE* genotyping

For apolipoprotein E (*APOE*) genotyping, DNA was isolated from 10 ml of ethylenediaminetetraacetic acid-preserved blood using the QIAamp DNA blood isolation kit from Qiagen (Hilden, Germany). The genotype was determined with the LightCycler *APOE* mutation detection kit (Roche Diagnostics GmbH, Mannheim, Germany).

### Parallel reaction monitoring MS panel

The sample preparation and acquisition methods for PRM analysis are described in detail elsewhere [29]. For the present study, we used the same sample preparation and MS methods, with minor modifications as described below. Because quantitative MS analysis of intact proteins is currently not feasible, a number of peptides suitable for MS analysis were obtained by digestion of CSF proteins with trypsin. Using nanoflow LC-MS in a shotgun approach, we injected 1 μl of trypsin-digested CSF from patients with and without AD biomarker profiles. Using this approach, we detected more than 2000 tryptic peptides from more than 400 proteins (unpublished data, G. Brinkmalm, Oct 2013). Based on these experiments and the current literature, a number of proteins were selected for further investigation. For each protein, two or three proteotypic peptides were selected. Corresponding stable isotope-labeled peptides were added and used as reference peptides in the PRM analyses. In addition, known amounts of bovine serum albumin (BSA) protein and a corresponding stable isotope-labeled peptide were added to each sample as a reference to monitor sample-processing variations for example digestion. To ensure good-quality data, the mass spectrometer was operated in a relatively slow mode with long injection times (up to 300 milliseconds) per acquisition. Because this limited the maximum number of peptides that could be handled simultaneously, we divided the proteins into two panels. The first panel included CysC, β_2_M, VGF, CHGA, and SCG2, and the second panel included LysC, NRXN-1, NRXN-2, NRXN-3, NPTX1, NFASC, and NCANP. The turnaround time was 72 minutes per sample, which made it impractical to analyze all data on one occasion. Therefore, we divided the dataset into three subsets (randomized according to diagnosis, age, and sex) and performed digestion as well as PRM analysis at different times. This introduced small variations in digestion and LC-MS performance (day-to-day variations). To adjust for these effects, a CSF pool was aliquoted, and four pool samples were used as calibrants in each run of 40 samples. Study samples, CSF pool samples, and heavy isotope-labeled standards were prepared as previously described [29], except that standard 0.5-, 1.5-, and 2.0-ml polypropylene tubes were used in the present work. After thawing, 20 μl of the internal standard mixture was added to each sample. Tryptic digestion was performed as previously described [29], except that no shaking of samples was performed. Digested samples were centrifuged, desalted using Oasis 30-μm HLB 96-well μElution plates (Waters, Milford, MA, USA) with MeOH for elution into polypropylene plates. Sample eluates were transferred into Eppendorf LoBind vials (Eppendorf, Hauppauge, NY, USA), dried in a SpeedVac (Thermo Fisher Scientific, Waltham, MA, USA), and stored at −80 °C pending PRM analysis. Directly prior to PRM analysis, samples were reconstituted in 50 mM NH_4_HCO_3_ and split as desired for each panel. Twenty-five microliters of CSF were used for panel 1, and 50 μl were used for panel 2.

PRM analysis was performed using a Dionex UltiMate 3000 system (SRD-3600 degasser, WPS-3000TPL autosampler, LPG-3600 pump, FLM-3100 column compartment; Thermo Fisher Scientific) coupled to a Thermo Q Exactive electrospray ionization hybrid quadrupole-Orbitrap high-resolution mass spectrometer (Thermo Fisher Scientific). Separation was performed with a Hypersil GOLD reversed phase column (inner diameter 2.1 mm, length 100 mm; Thermo Fisher Scientific) operated at a flow rate of 100 μl/minute. Mobile phase A was 0.1% formic acid, mobile phase B was 84% acetonitrile/0.1% formic acid (vol/vol), and the gradient length was 50 minutes. CVs of BSA in the CSF pool samples were (mean ± SD over the three runs) 2.3 ± 1.1% in the first PRM panel and 2.3 ± 1.4% in the second PRM panel. In the study samples, BSA showed CVs of 2.3 ± 0.5% for the first panel and 2.4 ± 0.1% for the second panel. Levels correlated highly with each other between the two PRM panels (Pearson’s *r* = 0.92) and did not differ between diagnostic groups, but they were slightly different between the subsets subjected to trypsinization and PRM analysis on separate occasions (*see* Additional file 1). CVs of the proteins of the PRM panel are described in detail elsewhere [29]. The CSF pool samples were used to normalize data between acquisition days. This was done individually for each peptide. Data processing was performed as described elsewhere [29].

### Statistical analysis

For statistical analysis, IBM SPSS Statistics version 22.0 for Windows software (IBM, Armonk, NY) was used. We assessed differences in patient characteristics using the chi-square test, Student’s *t* test, or analysis of variance when appropriate. Because CSF Aβ_42_, tau, and p-tau were not normally distributed, we logarithmically transformed them for these analyses. Because Aβ_42_ samples were collected over a long period, we rescaled values of Aβ_42_ according to recent insights from our group that Aβ_42_ levels show a significant drift over time [35]. Because of their closely related biological functions, most proteins of the PRM assay were expected to correlate with each other within individuals. Therefore, we used generalized estimating equations (GEEs) with an exchangeable correlation structure, and we clustered the peptides in two levels: all peptides within one subject and all peptides within one protein (i.e., within-subject variables). To achieve a similar order of magnitude in peptide levels, we transformed them to Z-scores for this analysis. Within this model, we analyzed differences of the proteins (entered as dependent variables) between diagnostic groups (entered as independent variables) using a multivariate linear model. To assess if there were differential effects of individual proteins, we included the diagnosis × protein interaction in the model. Analyses were adjusted for age, sex, *APOE* ε4 allele status (dichotomized into no ε4 alleles or at least one ε4 allele), and PRM run (to account for day-to-day variation). We recoded diagnostic groups to estimate effect sizes (ß) and SEs for different diagnostic groups. Bonferroni corrections were used to adjust *p* values for multiple comparisons. To assess which proteins differed between diagnostic groups, we subsequently performed the GEE analysis stratified for individual proteins. In this analysis, each protein (i.e., as a cluster of the peptides from that protein) was assessed in a separate model as a dependent variable. In addition, we repeated all GEE analyses including only the subjects with MCI to compare subjects with sMCI with subjects with MCI-AD. Patients progressing to another type of dementia (*n* = 3) were excluded from this analysis. Last, we assessed associations of the peptides with the established CSF AD biomarkers Aβ_42_, tau, and p-tau using Spearman’s correlations. Statistical significance was set at *p* < 0.05 for main effects and at *p* < 0.10 for interactions.

## Results

### Baseline characteristics

Table 1 shows the demographic and clinical characteristics of the study population according to baseline and follow-up diagnosis. Patient groups were well matched for age and sex. As expected, patients with AD dementia had the lowest MMSE values, and patients with MCI had values between those of patients with AD dementia and control subjects. CSF Aβ_42_ was lower, and total tau and p-tau levels were higher, in patients with AD dementia, whereas levels of patients with MCI were between those of patients with AD dementia and control subjects. In patients with MCI-AD, biomarker levels were similar to those of patients with AD dementia, and in patients with sMCI, Aβ_42_ and tau levels differed only slightly from those of control subjects, but all three biomarker levels were evidently different from those of patients with AD and patients with MCI-AD.

**Table 1:** Patient characteristics according to diagnosis

	Age, years	Sex, F/M (% female)	MMSE score at baseline	CSF Aβ_42_ (pg/ml)	CSF tau (pg/ml)	CSF p-tau (pg/ml)
Control subjects, *n* = 40	64.5 ± 8.2	19/21 (48%)	28.3 ± 1.7	1086 ± 161	228 ± 64	40 ± 9
MCI, *n* = 40	64.5 ± 8.1	19/21 (48%)	26.3 ± 2.3^*^	839 ± 297^*^	481 ± 256^*^	69 ± 33^*^
sMCI, *n* = 23	63.7 ± 7.4	11/12 (48%)	26.7 ± 2.0	941 ± 306^†^	338 ± 182^†^	51 ± 21
MCI-AD^a^, *n* = 14	65.6 ± 9.0	7/7 (50%)	25.9 ± 2.7^†^	632 ± 84^*,$^	722 ± 186^*,$^	99 ± 28^*,$^
AD dementia, *n* = 40	64.6 ± 8.1	19/21 (48%)	21.6 ± 3.9^*^	640 ± 91^*,‡,$^	740 ± 433^*,‡,$^	94 ± 47^*,‡,$^

*Abbreviations: Aβ*_*42*_ Amyloid-β 1–42, *AD* Alzheimer’s disease, *CSF* Cerebrospinal fluid, *MCI* Mild cognitive impairment, *MMSE* Mini Mental State Examination, *p-tau* Tau phosphorylated at threonine 181, *sMCI* Stable mild cognitive impairment

Data are displayed as mean ± SD unless otherwise indicated. Analysis of variance with post hoc Bonferroni corrections or chi-square tests were used when appropriate. CSF Aβ_42_, total tau, and p-tau were logarithmically transformed for the analyses because of skewed values; in the table, crude values are shown

^a^MCI patients progressing to another form of dementia (*n*=3) were excluded

^*^p < 0.01 vs controls

^†^*p* < 0.05 vs controls

^‡^*p* < 0.01 vs all MCI patients

^$^*p* < 0.01 vs sMCI patients

### Differences of proteins between diagnostic groups

**Fig. 1:**
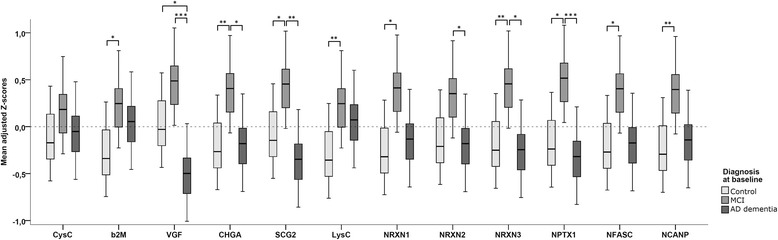
Mean adjusted Z-scores for individual proteins per diagnostic group. Shown are box plots of the adjusted Z-scores for individual proteins per diagnostic group as results derived from the GEE analysis with all peptide clusters and the diagnosis × protein interaction. *p* Values were derived from the analyses stratified according to protein, and pairwise comparisons were performed with post hoc Bonferroni corrections. Inherent to the definition of Z-scores, the mean of the total population is point 0 on the *y*-axis. *p < 0.05; **p < 0.01, ***p < 0.001. *AD* Alzheimer’s disease, *β*_*2*_*M* β_2_-Microglobulin, *CHGA* Chromogranin A, *CysC* Cystatin C, *LysC* Lysozyme C, *MCI* Mild cognitive impairment, *NCANP* Neurocan core protein, *NFASC* Neurofascin, *NPTX1* Neuronal pentraxin 1, *NRXN* Neurexin, *SCG2* Secretogranin 2, *VGF* Neurosecretory protein VGF

**Fig. 2:**
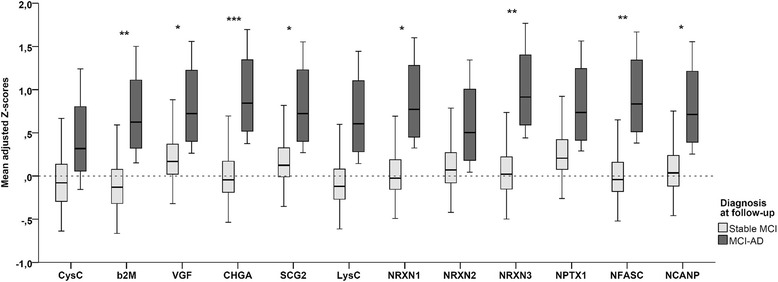
Mean adjusted Z-scores for individual proteins in patients with stable MCI and patients with MCI-AD. Shown are box plots of the adjusted Z-scores for individual proteins per diagnostic group as results from the GEE analysis with all peptide clusters and the diagnosis × protein interaction. *p* Values were derived from the analyses stratified according to protein. *p < 0.05; **p < 0.01, ***p < 0.001. *β*_*2*_*M* β_2_-Microglobulin, *CHGA* Chromogranin A, *CysC* Cystatin C, *LysC* Lysozyme C, *MCI* Mild cognitive impairment, *MCI-AD* Mild cognitive impairment progressing to Alzheimer’s disease dementia, *NCANP* Neurocan core protein, *NFASC* Neurofascin, *NPTX1* Neuronal pentraxin 1, *NRXN* Neurexin, *SCG2* Secretogranin 2, *VGF* Neurosecretory protein VGF

GEE analysis showed a main effect for diagnosis (Wald chi-square = 11.7, *p* = 0.003), as well as an interaction between diagnosis and protein (*p* < 0.001), indicating that the effect for diagnosis differed per protein. Analyses stratified according to protein showed main effects for diagnosis in all proteins but CysC (Fig. 1). Most proteins were elevated in the MCI group, and effect sizes were largest for CHGA (β = 0.63 [SE 0.17] vs control subjects and 0.54 [0.23] vs AD), SCG-2 (β = 0.53 [SE 0.20] vs control subjects and 0.74 [SE 0.22] vs AD), NRXN-3 (β = 0.60 [SE 0.20] vs control subjects and β = 0.65 [SE 0.22] vs AD), and NPTX1 (β = 0.63 [SE 0.22] vs control subjects and β = 0.78 [SE 0.21] vs AD). VGF showed a different pattern with evidently lower values in patients with AD dementia than in control subjects (β = −0.46 [SE 0.19]) and in patients with MCI (−0.93 [SE 0.22]), whereas the difference between patients with MCI and control subjects was only borderline significant (β = 0.47 [SE 0.20], *p* = 0.05). In the GEE analysis comparing patients with sMCI with patients with MCI-AD, there was a main effect for diagnosis as well (Wald chi-square = 6.55, *p* = 0.02). The overall interaction between diagnosis and protein was just above the level of significance (*p* = 0.12); however, to be able to compare this analysis with the one with baseline diagnosis, we also stratified this analysis according to protein. This showed main effects for most proteins, except for CysC, LysC, NRXN-2, and NPTX1. The other proteins were elevated in the subjects with MCI-AD compared with the subjects with sMCI, with the largest effect sizes being for CHGA (β = 0.98 [SE 0.26]), NRXN-3 (β = 0.96 [SE 0.35]), and NFASC (β = 0.92 [SE 0.35]). Results of this analysis are shown in Fig. 2.

### Correlation with CSF AD biomarkers

Correlations using Spearman’s rank correlation showed that most peptides, except those from LysC, correlated with CSF tau and p-tau in each diagnostic group (*see* Additional file 2). Generally, in the MCI-AD and AD groups, the correlations with p-tau were stronger than those with tau, whereas in control subjects and subjects with sMCI, tau and p-tau correlated similarly with most peptides. Correlations were stronger between (p)tau and the NRXNs, NPTX1, NFASC, and NCANP than those between (p)tau and the other peptides. Within control subjects and patients with sMCI, most peptides correlated positively with Aβ_42_, whereas they tended to be negative in patients with MCI-AD. Among patients with AD, there were no correlations with Aβ_42_.

## Discussion

In this study, we aimed to validate a PRM-MS assay consisting of a panel of proteins presumed to be involved in processes regarding secretory vesicle functioning, synaptic functioning, and innate immunity [12–21]. Having performed a pilot study [29], in the present study, we investigated this panel in a larger and independent cohort that included subjects with MCI in addition to patients with AD dementia and control subjects.

Contrary to our expectations, levels of most proteins were higher in patients with MCI than in the other groups, a finding attributable mainly to those patients who at follow-up had progressed to AD dementia. Stratified analyses showed that these differences were driven mainly by proteins involved in secretory vesicle function (SCG2, CHGA) or synapse formation and stabilization (NRXNs, NPTX1, NCANP), whereas CysC (a cysteine protease inhibitor) was similar between groups and LysC and β_2_M (both involved in the immune system) showed only small, borderline significant differences. In addition, we found that correlations of most PRM peptides with Aβ_42_ differed markedly between groups, with positive correlations in control subjects and subjects with sMCI but negative correlations in subjects with MCI-AD. In contrast to the findings of our pilot study [29], there were no differences between patients with AD dementia and control subjects, except for VGF. Importantly, however, the proteins showing differences between groups in the present study were the same proteins as in the pilot study. Hence, the difference between the two studies may be due to differences in the selection of patients or control subjects.

The results regarding VGF in the present study are in line with our pilot study, with clearly lower values in AD dementia than in the other groups. In the biomarker panel in the present study, VGF has been studied most extensively in AD research. Lower levels in AD dementia than in control subjects have been described before [13, 24, 28, 36]. In experiments with hippocampal neurons from rats, VGF peptides increased synaptic plasticity and enhanced proliferation of cells [37, 38]. Moreover, in another study, VGF-knockout mice showed memory impairment [39]. Our findings of lower levels in patients with AD dementia are hence concordant with these previous studies.

To our knowledge, increased levels of synaptic proteins in patients with MCI due to AD have not been described before. However, on one hand, a recent study showed that higher levels of CHGA in CSF predict future decreases in Aβ_42_ in healthy elderly subjects [25]. On the other hand, a decreased level in brain tissue and CSF of patients with AD compared with control subjects has also been described [27, 28, 40]. Studies on the role of NRXNs, NPTX1, NFASC, and NCANP in AD are rare, although the proteins have all been suggested to have a role in synapse formation, plasticity, and stability [17–21]. On the basis of using MS-based proteomics, CSF NRXN-1 has been found to be slightly decreased in patients with AD compared with control subjects in a small cohort of 16 subjects [36]. A recent study in mice suggested that NRXN-2 interacts with Aβ oligomers, resulting in loss of synapses, whereas blocking of this interaction prevented Aβ-induced memory impairment [41]. NPTX1 has been found to increase in cell cultures after treatment with Aβ, to be present in dystrophic neurites and around Aβ plaques in AD brains, and to modulate synaptic transmission [42, 43]. A recent study showed that NCANP expression in astrocytes was increased after incubation with Aβ [44]. It has been established that synaptic damage and loss are early events in AD that are already evident in the MCI stage [5]. Moreover, studies in the early 1990s showed that although the number of synapses declined in early stages of AD, this was first compensated by an increase in synapse size of the remaining synapses and only in later stages was overcome by further synapse loss [45, 46]. Our finding that the correlations between PRM peptides and Aβ_42_ differed between disease stages also suggests a dynamic pathophysiological trajectory with progression of the disease. The positive correlations in control subjects and subjects with stable MCI suggest an association with normal aging, whereas this association is reversed in cases of AD pathology. It can be hypothesized that a compensatory mechanism in patients in an early stage of AD pathology results in upregulation of proteins involved in synaptic plasticity and stabilization, whereas concentrations decline in later stages, associated with further synapse loss. Studies finding increased connectivity using functional MRI in amyloid-positive elderly subjects may also point in this direction [47, 48]. Because our AD dementia group consisted of patients still in a relatively early stage of the disease, concentrations at the time of measurement may have been between this elevated level and a subsequent decrease. This was a cross-sectional study, however, and longitudinal studies with extensive clinical follow-up and repeated lumbar punctures are needed to assess changes of these proteins within individuals over the course of the disease. Subjects in the earliest disease stages (SCD and MCI at baseline) will be most interesting to investigate for further validation of the present study.

Among the strengths of our study are the relatively large number of patients for this type of experiment and the fact that the cohort was matched for sex and age. The small size of the group with MCI due to AD was a limitation, however, and the results have to be replicated in independent cohorts to be able to draw definitive conclusions. The use of patients with subjective cognitive decline as control subjects in our study is a possible limitation. The advantage, however, is that these subjects received the same cognitive screening as the other patients, ruling out other diagnoses with as much certainty as possible. In addition, the aim was to find CSF biomarkers useful for routine clinical practice; therefore, using perfectly healthy control subjects instead of subjects with SCD might overestimate differences. Strengths of the PRM technique are that it allows for investigation of proteins for which there are no antibodies available and that the specificity is very high. This is important in the search for new biomarkers and for verification of shotgun proteomic findings. A disadvantage, however, is the time needed for analysis, which makes high throughput of a large volume of samples, as would be needed in daily practice, unfeasible.

## Conclusions

Our results suggest that several proteins involved in vesicular transport and synaptic stability are elevated in patients with MCI, especially in patients with MCI progressing to AD dementia. This may reflect early events in the AD pathophysiological cascade. There were no differences between control subjects and subjects with AD, suggesting that the proteins are probably not suitable as diagnostic biomarkers. However, these proteins may be useful as disease stage or prognostic markers in an early symptomatic stage of the disease, in addition to the core diagnostic markers Aβ_42_ and tau. Whether our findings are consistent, as well as whether these proteins could indeed be used for prognosis, monitoring of disease progression, or assessment of effect of therapy, is a subject for further study.

## Additional files

Additional file 1: Figure S1. Levels of bovine serum albumin per diagnostic group and PRM run. Shown are dot plots of bovine serum albumin added as a reference peptide to monitor sample-processing variations. (PDF 201 kb)

Additional file 2: Table S1. Correlations of PRM peptides with CSF AD biomarkers. Shown are results of Spearman’s rank correlations between PRM peptides and CSF AD biomarkers in each diagnostic group. (DOCX 22 kb)

## Abbreviations

AD: Alzheimer’s disease
APOE: Apolipoprotein E
Aβ: Amyloid-β
Aβ_42_: Amyloid-β 1–42
BSA: Bovine serum albumin
CHGA: Chromogranin A
CSF: Cerebrospinal fluid
CV: Coefficient of variation
CysC: Cystatin C
GEE: Generalized estimating equation
LysC: Lysozyme C
MCI: Mild cognitive impairment
MCI-AD: Mild cognitive impairment progressing to Alzheimer’s disease dementia
MMSE: Mini Mental State Examination
MRI: Magnetic resonance imaging
NCANP: Neurocan core protein
NFASC: Neurofascin
NPTX1: Neuronal pentraxin 1
NRXN: Neurexin
PRM: Parallel reaction monitoring
p-tau: Tau phosphorylated at threonine 181
SCD: Subjective cognitive decline
SCG2: Secretogranin 2
sMCI: Stable mild cognitive impairment
VGF: Neurosecretory protein VGF
β_2_M: β_2_-Microglobulin
