# Supplementary figures and images for: Synaptic proteins in CSF as potential novel biomarkers for prognosis in prodromal Alzheimer’s disease
Source: Alzheimers Res Ther. 2018 Jan 15;10:5. doi: 10.1186/s13195-017-0335-x (PMC6389073; doi:10.1186/s13195-017-0335-x)

**Figure S1 Levels of bovine serum albumin per diagnostic group and PRM run**

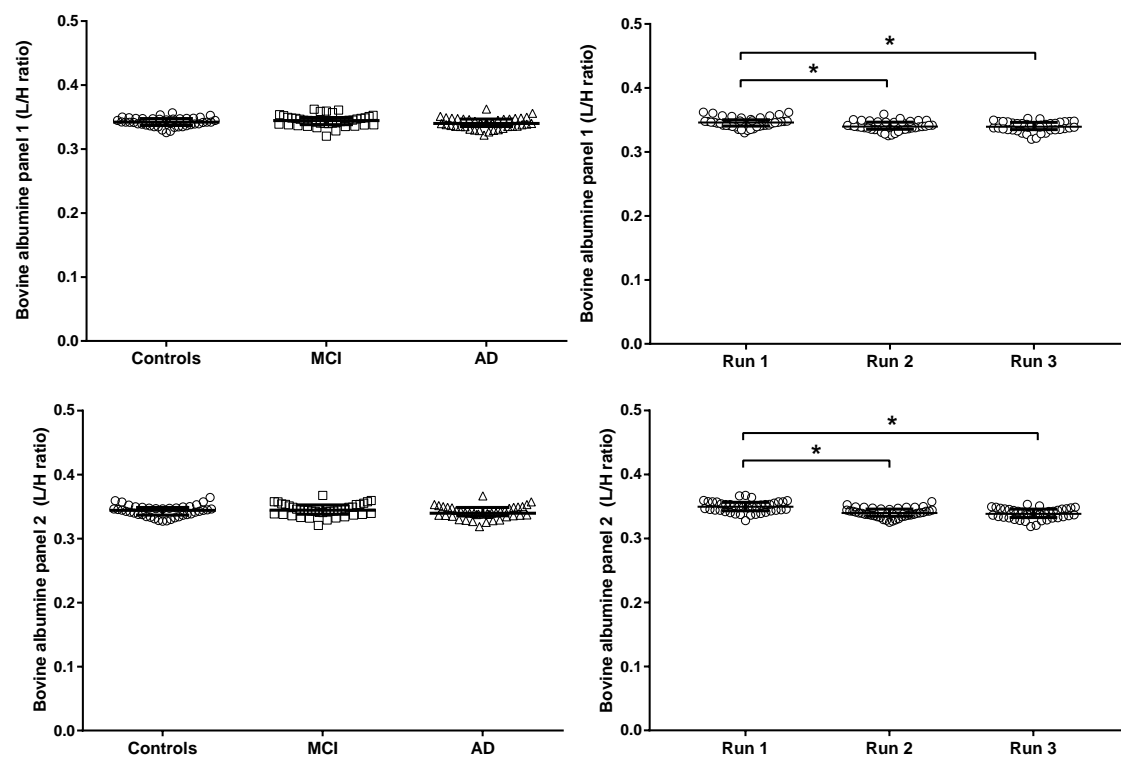

\*  $p < 0.01$

Supplement: Supplementary file 1 — Levels of bovine serum albumin per diagnostic group and PRM run. Shown are dot plots of bovine serum albumin added as a reference peptide to monitor sample-processing variations. (PDF 201 kb) [file 13195_2017_335_MOESM1_ESM.pdf]
