# Supplementary material for: Synaptic proteins in CSF as potential novel biomarkers for prognosis in prodromal Alzheimer’s disease
Source: Alzheimers Res Ther. 2018 Jan 15;10:5. doi: 10.1186/s13195-017-0335-x (PMC6389073; doi:10.1186/s13195-017-0335-x)
Supplement: Supplementary file 2 — Correlations of PRM peptides with CSF AD biomarkers. Shown are results of Spearman’s rank correlations between PRM peptides and CSF AD biomarkers in each diagnostic group. (DOCX 22 kb) [file 13195_2017_335_MOESM2_ESM.docx]

|  |  | **Controls (n=40)** | | | **Stable MCI (n=23)** | | | **MCI-AD (n=14)** | | | **AD dementia (n=40)** | | |
| --- | --- | --- | --- | --- | --- | --- | --- | --- | --- | --- | --- | --- | --- |
| **Protein** | **Peptide sequence** | **CSF Aβ42** | **CSF tau** | **CSF p-tau** | **CSF Aβ42** | **CSF tau** | **CSF p-tau** | **CSF Aβ42** | **CSF tau** | **CSF p-tau** | **CSF Aβ42** | **CSF tau** | **CSF p-tau** |
| **Cystatin-C** | LVGGPMDASVEEEGVR | 0.52** | 0.64** | 0.58** | -0.07 | 0.43* | 0.54** | -0.01 | 0.23 | 0.38 | -0.05 | 0.43** | 0.50** |
|  | ALDFAVGEYNK | 0.40** | 0.72** | 0.61** | 0.56** | 0.52* | 0.58** | -0.09 | 0.29 | 0.52^#^ | 0.05 | 0.50** | 0.56** |
| **B2-microglobuline** | VEHSDLSFSK | 0.43** | 0.50** | 0.58** | -0.07 | 0.46* | 0.48* | 0.03 | 0.13 | 0.44 | -0.12 | 0.18 | 0.23 |
|  | VNHVTLSQPK | 0.18 | 0.71** | 0.60** | 0.43* | 0.53** | 0.52* | 0.03 | 0.30 | 0.53* | 0.02 | 0.24 | 0.33* |
| **Neurosecr. protein VGF** | NSEPQDEGELFQGVDPR | 0.43** | 0.63** | 0.48** | 0.58** | 0.57** | 0.52* | -0.18 | 0.36 | 0.62* | -0.14 | 0.52** | 0.56** |
|  | AYQGVAAPFPK | 0.41** | 0.61** | 0.45** | 0.56** | 0.60** | 0.66** | -0.34 | 0.35 | 0.67** | -0.13 | 0.51** | 0.57** |
| **Chromogranin-A** | YPGPQAEGDSEGLSQGLVDR | 0.60** | 0.65** | 0.49** | 0.15 | 0.44* | 0.54** | 0.09 | 0.35 | 0.46^#^ | -0.20 | 0.38* | 0.44** |
|  | GLSAEPGWQAK | 0.34* | 0.68** | 0.47** | 0.54** | 0.52* | 0.61** | 0.01 | 0.34 | 0.57* | -0.11 | 0.50** | 0.57** |
|  | EDSLEAGLPLQVR | 0.09 | 0.57** | 0.24 | 0.56** | 0.43* | 0.39 | -0.02 | 0.22 | 0.47^#^ | -0.03 | 0.38* | 0.45** |
| **Secretogranin-2** | ALEYIENLR | 0.40* | 0.73** | 0.53** | 0.55** | 0.58** | 0.64** | -0.17 | 0.43 | 0.71** | -0.03 | 0.51** | 0.58** |
|  | VLEYLNQEK | 0.39* | 0.73** | 0.53** | 0.64** | 0.54** | 0.56** | -0.24 | 0.44 | 0.76** | -0.03 | 0.43** | 0.54** |
| **Lysozyme-C** | WESGYNTR | 0.02 | 0.47** | 0.27 | 0.44* | 0.02 | 0.09 | -0.31 | -0.00 | 0.28 | 0.08 | 0.00 | 0.03 |
|  | STDYGIFQINSR | -0.06 | 0.47** | 0.28 | 0.48* | -0.03 | -0.01 | -0.30 | -0.16 | 0.15 | 0.16 | -0.06 | -0.04 |
| **Neurexin-1** | LTVDDQQAMTGQMAGDHTR | 0.43** | 0.63** | 0.65** | 0.45* | 0.63** | 0.61** | -0.42 | 0.40 | 0.74** | 0.03 | 0.50** | 0.53** |
|  | VDSSSGLGDYLELHIHQGK | 0.34* | 0.53** | 0.55** | 0.46* | 0.60** | 0.58** | -0.28 | 0.32 | 0.62* | -0.09 | 0.57** | 0.62** |
| **Neurexin-2** | TALAVDGEAR | 0.38* | 0.55** | 0.61** | 0.51* | 0.58** | 0.60** | -0.33 | 0.34 | 0.50^#^ | 0.07 | 0.47** | 0.48** |
|  | VDLPLPPEVWTAALR | 0.58** | 0.60** | 0.56** | 0.17 | 0.57** | 0.65** | -0.50^#^ | 0.18 | 0.55* | -0.07 | 0.46** | 0.42** |
| **Neurexin-3** | FI(C)D(C)TGTGYWGR | 0.37* | 0.64** | 0.56** | 0.62** | 0.55** | 0.61** | -0.27 | 0.31 | 0.65* | -0.02 | 0.54** | 0.60** |
|  | LTVDDDVAEGTMVGDHTR | 0.47** | 0.65** | 0.60** | 0.48* | 0.52* | 0.58** | -0.39 | 0.30 | 0.69** | -0.12 | 0.60** | 0.65** |
| **Neuronal pentraxin-1** | LENLEQYSR | 0.35* | 0.50** | 0.47** | 0.41 | 0.49* | 0.46* | -0.55* | 0.27 | 0.50^#^ | 0.01 | 0.44** | 0.50** |
|  | LTPGEVYNLAT(C)STK | 0.38* | 0.59** | 0.51** | 0.46* | 0.61** | 0.56** | -0.35 | 0.31 | 0.65* | -0.01 | 0.44** | 0.50** |
| **Neurofascin** | GNPAPSFHWTR | 0.33* | 0.67** | 0.54** | 0.58** | 0.57** | 0.64** | -0.35 | 0.28 | 0.60* | -0.05 | 0.51** | 0.60** |
|  | VIAINEVGSSHPSLPSER | 0.31* | 0.71** | 0.57** | 0.61** | 0.58** | 0.62** | -0.25 | 0.25 | 0.60* | -0.08 | 0.57** | 0.60** |
| **Neurocan core protein** | ELGGEVFYVGPAR | 0.31* | 0.64** | 0.49** | 0.53** | 0.60** | 0.63** | -0.48 | 0.27 | 0.59* | -0.07 | 0.51** | 0.58** |
|  | DFQWTDNTGLQFENWR | 0.21 | 0.69** | 0.48** | 0.48* | 0.71** | 0.70** | -0.28 | 0.40 | 0.73** | -0.07 | 0.47** | 0.53** |

**Table S1** Correlations of PRM peptides with CSF AD biomarkers

^#^ p < 0.10, * p < 0.05, ** p < 0.01

Correlations were performed using crude values of the analytes and Spearman rank correlation. Shown are *r*’s for every peptide of each protein.
